# Research on casing damage risk early warning technology based on MIT-MTT logging and 16S rRNA gene analysis

**DOI:** 10.3389/fmicb.2025.1708148

**Published:** 2025-12-15

**Authors:** Shuoliang Wang, Shiqi Wang, Congcong Li, Changhao Zhou, Liangliang Jiang

**Affiliations:** 1 School of Energy, Faculty of Engineering, China University of Geosciences, Beijing, China; 2 College of Civil Engineering and Architecture, Shandong University of Aeronautics, Binzhou, Shandong, China; 3 Department of Chemical and Petroleum Engineering, University of Calgary, Calgary, AB, Canada

**Keywords:** casing damage, MIT-MTT integrated logging technology, 16S rRNA gene analysis, sulfate-reducing bacteria, risk early warning

## Abstract

With the continuous advancement of oilfield development, the number of oil–water wells experiencing casing damage due to corrosion has increased annually, which has seriously affected the injection-production balance and significantly reduced the potential for recovering residual oil. Because the water cut trends in casing-damaged wells are similar to those in water breakthrough injection wells, identifying wells with casing damage has been challenging. This study is the first to combine the Multi-Finger Imaging Tool (MIT)-Magnetic Thickness Tool (MTT) integrated logging technology with 16S rRNA gene analysis to systematically analyze the relationship between casing damage and the composition of microbial communities. The results indicated that there were significant differences in the high-corrosion zones at various depths, and the structure of the sulfate-reducing bacterial community in the produced fluids also varied. In particular, in deeper zones, the relative abundance of thermophilic bacteria, represented by Thermotogata, increased significantly. Moreover, the more severe the casing damage, the more dominant the sulfate-reducing bacteria became in the microbial community of the produced fluids. After secondary sealing treatment, the proportion of sulfate-reducing bacteria was significantly reduced. The study further found that sulfate-reducing bacteria primarily belonged to the phyla Proteobacteria, Bacillota, Thermotogata, and Thermodesulfobacteriota, while significant populations of iron-reducing bacteria were not detected in the produced fluids. This finding suggests that sulfate-reducing bacteria are the main microbial factor causing metal corrosion of the casings. Innovatively, this study proposes a biometal corrosion monitoring method for production wells based on microbial community structure, thereby providing a novel technical approach to preventing oil well corrosion.

## 1 Introduction

During oilfield development, as oil and gas recovery rates continue to increase, the service environment for casings becomes increasingly harsh. The downhole environment is complex and variable, encompassing multiple fluid media, while temperature and pressure conditions fluctuate continuously, resulting in dynamic changes in the metabolic activities of microbial communities. These factors collectively act upon steel casings, leading to varying degrees of corrosion. In extreme cases, oil well casings may experience issues such as buckling, deformation, perforation, and fissure rupture, thereby exacerbating casing damage (Wang et al., 2022; Wang et al., 2023). Related studies have demonstrated that corrosion is one of the primary causes of casing damage, accounting for over one-third of all wells exhibiting casing failures (Wasim and Djukic, 2022). This problem not only results in significant economic losses but also may lead to safety incidents and environmental pollution, posing a serious threat to the sustainable development of oilfields. Therefore, a comprehensive investigation into the factors and mechanisms of casing corrosion during oilfield development is of paramount importance for developing effective protective measures, extending casing service life, ensuring safe oilfield production, and promoting environmental protection; this has consequently become a focal topic of current research.

During crude oil extraction, casings are particularly susceptible to internal corrosion. This process is influenced by a combination of factors including the material of the tubing, the position of the dynamic liquid level, the composition of the produced water medium, flow velocity, pH, temperature, types and concentrations of corrosive gasses, and various environmental parameters such as microbial activity. Temperature and microorganisms play especially critical roles; in specific sections of the tubing, they are key factors that lead to severe corrosion (Wei et al., 2022). Microbiologically influenced corrosion (MIC) refers to the process in which microorganisms attach to the surfaces of heterogeneous materials, forming biofilms and bacterial aggregates whose metabolic activities directly or indirectly accelerate the corrosion reactions (Victoria et al., 2021). Iron-oxidizing bacteria (IOB), iron-reducing bacteria (IRB), methanogenic archaea, acid-producing bacteria (APB), and sulfate-reducing bacteria (SRB) are the primary corrosive agents in pipelines within oil reservoirs or marine environments. According to relevant investigations, approximately 20% of the economic losses caused by corrosion are associated with MIC, of which SRB-induced corrosion accounts for about 70% of these events (Su et al., 2024). Previous studies have shown that knocking out the genes encoding hydrogenases and heme-containing cytochromes of SRB can reduce the corrosion rate of iron (Tripathi et al., 2021). SRB, which are anaerobic bacteria, are widely distributed in low-oxygen environments such as oil and gas fields and underground pipelines, exerting a significant impact on metal corrosion. Among corrosion studies, SRB have been the most extensively investigated, and their metabolic activities and biofilm formation have a dual role in metal corrosion (Jia et al., 2019). On one hand, through chemical MIC (CMIC), SRB can utilize sulfate, sulfite, thiosulfate, or even elemental sulfur present in the environment as electron acceptors, thereby producing H_2_S through their metabolic processes (Barton and Fauque, 2009). The generation of H_2_S and its accumulation within biofilms is a key factor leading to localized corrosion, posing significant threats to the integrity and safety of pipelines, and potentially triggering severe incidents such as leaks and explosions. On the other hand, under conditions of limited carbon sources, SRB can directly extract electrons from metal surfaces to sustain their metabolism, a process known as extracellular electron transfer MIC (EET-MIC), which accelerates metal corrosion and can particularly induce pitting corrosion. When sediments coexist with microorganisms on the pipe wall, this corrosion effect is further amplified. The growth of microorganisms within the microenvironment beneath the sediment can markedly increase the severity of localized corrosion, thereby posing a serious threat to pipeline integrity (Hou et al., 2024).

Microbiologically influenced corrosion is a complex form of corrosion involving the synergistic effects of multiple bacterial strains (Xu et al., 2023). The dynamic nature of microbial induced corrosion is due to its complex microbial community interactions, so tailored management methods are needed. However, many studies are limited to the influence of a single or a few strains, which is very different from the corrosion phenomenon observed in the field (Li et al., 2025). Although laboratory corrosion simulation experiments are helpful in studying the fundamental mechanisms of MIC, they differ substantially from the real environments that pipelines and similar materials are exposed to. Consequently, some studies may not accurately reflect the complex corrosion processes induced by mixed microbial communities *in situ*. In contrast, the combination of Multi-Finger Imaging Tool (MIT) and Magnetic Thickness Tool (MTT) integrated logging technologies can capture the real state of casing corrosion in underground environments. The MIT can detect changes in the inner diameter of the casing, while the MTT can measure variations in the casing wall thickness. Using the combined MIT–MTT technology for casing integrity evaluation enables the recording of multiple independent wellbore diameter curves, 12 magnetic wall thickness curves, as well as well deviation and relative azimuth curves in a single downhole operation. This data can be used to construct a three-dimensional image of the oil and casing, which visually represents the location and extent of corrosion, deformation, and perforations (Great, 2009; Yan et al., 2022). Furthermore, the application of high-throughput sequencing techniques assists in determining the composition and dynamic changes of the microbial communities involved in the corrosion process. For instance, Wei et al. (2025) used 16S rRNA gene analysis to reveal that the dominant microorganisms, Desulfomicrobium and Methanosarcinaceae, in nearshore produced water at 60 °C generated sulfides and methane, thereby promoting metal corrosion. Similarly, Reddy et al. studied metal corrosion in freshwater environments and, through 16S rRNA gene sequencing, discovered the enrichment of SRB and acetogenic bacteria, indicating a syntrophic relationship between them.

This study establishes a dual-analysis system based on “physical diagnostics–biological detection” to systematically elucidate the coupling mechanism between casing damage and microbial communities. At the engineering scale, the MIT–MTT integrated logging technique enabled three-dimensional positioning and quantitative characterization of casing corrosion defects, yielding key parameters such as corrosion hotspot distribution and corrosion severity. At the microbial scale, produced fluid samples were analyzed via 16S rRNA gene high-throughput sequencing to resolve the microbial community structure, and functional annotation identified the distribution of corrosion-related functional microbial groups such as SRB. By integrating the results from both techniques, a correlation analysis was performed between the condition of casing damage and the microbial community in the produced fluid. This work provides the theoretical foundation for establishing a microbial–corrosion coupling prediction model, contributing to the development of a predictive technology for pipeline corrosion based on microbial community profiling, and facilitating a shift in production strategy from “reactive maintenance” to “proactive prediction.”

## 2 Materials and methods

### 2.1 Sample source and processing

Samples were collected from the Taibai Liang operational area of the Changqing Oilfield (Table 1). Produced water was obtained from six casing-damaged wells (CDW1, CDW2, CDW3, CDW4, CDW5, CDW6; untreated with secondary sealing) and six wells after casing repair (PCDTW1, PCDTW2, PCDTW3, PCDTW4, PCDTW5, PCDTW6; treated with secondary sealing). The secondary sealing process involved setting a bridge plug to isolate the damaged sections of the casing. The produced liquid sample was aseptically collected at the wellhead using a sterile bag, with a volume of approximately 2 liters. Following collection, the sample was transported to the laboratory under cold chain conditions at −18 °C. Water sample analyses followed the SY/T5523-2016 “Oilfield Water Analysis Methods,” and ion concentrations were determined using an ion chromatograph (ICS-500). Sulfide content was measured with the methylene blue spectrophotometric method using a UH4150 ultraviolet–visible–near infrared spectrophotometer (Hitachi, Japan). SRB content was tested and analyzed by dilution-to-extinction method, according to the standard “Water quality specification and practice for analysis of oilfield injection water in clastic reservoirs” (SY/T 5329–2022). In order to avoid environmental pollution affecting the sequencing results, a blank control experiment was set up (Supplementary Table S1).

**Table 1:** Sample information.

Well Name	Production zone	pH	Depth (m)	Temperature (°C)
CDW1	Yan 10	6.6	2005	54
CDW2		6.5	2,190	59
CDW3		7.1	2072	55
CDW4		6.9	2050	56
CDW5		6.5	2,170	59
CDW6		6.3	2,108	58
PCDTW1		6.7	2,150	58
PCDTW2		8.2	2,120	57
PCDTW3		7.8	2,200	60
PCDTW4		7.3	2085	57
PCDTW5		8.1	2020	52
PCDTW6		7.6	2,135	59

### 2.2 DNA extraction and sequencing analysis

One liter of the aqueous phase was subjected to vacuum filtration using a 0.22-μm polycarbonate filter membrane. The resulting filter membrane was air-dried at room temperature, cut into 1-mm^2^ squares with sterile scissors, and stored at −80 °C for later use.

The samples were aliquoted into centrifuge tubes, to which 670 μL of lysozyme (50 mg/mL) was added. The mixture was incubated at 37 °C for 15 min to lyse the cell walls. Subsequently, 340 μL of proteinase K (20 mg/mL) and 3.4 mL of 20% SDS were added, and the mixture was incubated at 62 °C for 45 min with agitation every 9 min. The centrifuge was pre-cooled to 4 °C. The mixture was then equally divided into several tubes and centrifuged at 4 °C for 10 min at 12000 g. The supernatant was collected, to which LPA (5 mg/mL), sodium chloride, and isopropanol were added to precipitate DNA. The mixture was adjusted to volume with sterile water, incubated on ice at −20 °C for 40 min, and then centrifuged at 4 °C for 20 min at 12000 g to collect the precipitate. The precipitate was washed with 25 mL of 70% ethanol and centrifuged at 4 °C for 20 min at 12000 g to remove the supernatant. The pellet was resuspended in sterile water, transferred to a new centrifuge tube, and treated with phenol:chloroform:isoamyl alcohol. The mixture was centrifuged at room temperature for 15 min at 16000 g. The aqueous phase was transferred to a new tube, to which 0.04 volumes of 5 M NaCl and 2 volumes of absolute ethanol were added. The mixture was incubated on ice at −20 °C for 40 min and then centrifuged at 4 °C for 10 min at 14000 g to collect the precipitate. The pellet was washed with 1.2 mL of 70% ethanol and centrifuged at 4 °C for 5 min at 14000 g, repeating this step once. Finally, the pellet was dissolved in 50 μL of TE buffer, incubated at 65 °C for 5 min, and the supernatant was collected by centrifugation (Goldenberger et al., 1995). The supernatants were pooled and the DNA was purified.

The concentration and purity of the extracted DNA were determined using a NanoDrop 2000c spectrophotometer. Samples meeting the quality criteria were sent to Paisenno Biotechnologies for amplification of the V4–V5 hypervariable regions of the 16S rRNA gene, with bacterial primers 338F (5′-GTGYCAGCMGCCGCGGTAA-3′) and 806R (5′-CCGYCAATTYMTTTRAGTTT-3′) (Caporaso et al., 2011), followed by paired-end sequencing on an Illumina MiSeq platform. Microbiome bioinformatics analysis was performed using QIIME 22019.4 (Bokulich et al., 2018). Raw sequence data were processed with the demux plugin for demultiplexing, and the cutadapt plugin for primer removal. Quality filtering, denoising, merging, and chimera removal were conducted using the DADA2 plugin (Callahan et al., 2016). The resulting sequences were clustered at 100% similarity to generate amplicon sequence variants (ASVs) and an abundance table. Taxonomic assignment was performed using the Silva database (Release 132) (Quast et al., 2013). Prior to further analysis, sample data were rarefied. Alpha and beta diversity analyses, principal coordinate analysis (PCoA), hierarchical clustering, and linear discriminant analysis effect size (LEfSe) were performed using QIIME 22019.4 and R version 4.3.2 to evaluate bacterial communities in rock fragments at different depths. The microbial name was queried on the National Center for Biotechnology Information (NCBI) database to reconstruct the phylogenetic tree, which was subsequently visualized with iTOL (Interactive Tree Of Life) v7.2.2 (Letunic and Bork, 2024).

### 2.3 Functional prediction

The FAPROTAX database^1^ was employed to predict the community functions of prokaryotes in the oil–water samples (Louca et al., 2016). The previously compiled ASV abundance table was compared with the groups available in the FAPROTAX database, yielding annotations related to the major processes and nutritional types involved in the biogeochemical cycling of carbon, hydrogen, nitrogen, sulfur, and other elements—for example, respiration, methanogenesis, sulfur oxidation, nitrogen fixation, nitrification, and denitrification—covering 90 functional groups of prokaryotes. Additionally, the BugBase database^2^ was used to predict microbial phenotypes such as oxygen tolerance and oxidative stress resistance (Ward et al., 2017).

### 2.4 Principles of MIT and MTT logging

The MIT instrument is available in various configurations with 24, 40, or 60 arms (Table 2). Its operating principle is based on the fact that variations in the casing inner diameter cause the measurement arms to extend or retract. As the tips of the measurement arms move radially relative to the instrument, a conversion mechanism translates this movement into a vertical displacement, which is then transmitted to a magnetic core within a displacement sensor. This displacement alters the position of the magnetic core relative to the induction coil within the sensor.

**Table 2:** Instrument parameters for MIT and MTT logging.

Instrument parameters	MIT			MTT
Number of caliper arms	24	40	60	
Outer diameter (mm)	43	70	102	43
Length (m)	1.14	1.66	1.75	2.12
Mass (kg)	9.1	28.0	45.0	13.6
Pressure rating (psi)	15,000			
Temperature Rating (°C)	150			
Measuring range (mm)	45 ~ 114	76.2 ~ 190.5	114 ~ 245	50.8 ~ 177.8
Vertical resolution (mm)	2.54	2.54	2.54	Metal loss rate >35%, wall thickness loss >50%, holes >10 mm identifiable; Metal loss rate >20%, wall thickness loss >30%, holes >20 mm identifiable.
Radial resolution (mm)	0.076	0.127	0.178	

The MTT electromagnetic thickness tool is equipped with 13 coils, consisting of one excitation coil and 12 receiving coils. An alternating current passing through the excitation coil generates a magnetic field that couples with the casing and the receiving coils. The phase lag of the induced signal in the receiving coils relative to the excitation current is directly proportional to the average wall thickness of the casing. For casings with a constant diameter, a thicker wall results in a larger phase shift. The MTT leverages the relationship between the phase angle of the induced electromotive force and the casing wall thickness for its measurements.

## 3 Results and discussion

### 3.1 Corrosion classification

In the main body of the casing, a wall thickness loss of 0.51 mm or more is defined as corrosion. For the connection sections, corrosion is defined when the wall thickness loss reaches 0.76 mm or more. The degree of metal corrosion is calculated as the percentage of the average corrosion loss of an individual casing relative to its standard wall thickness (Table 3).

**Table 3:** Classification of corrosion types.

Corrosion level	Description	Degree of corrosion
Mild corrosion	Slight corrosion	Less than 20%
Moderate corrosion	Moderately severe corrosion	20–40%
Severe corrosion	Severe corrosion	40–85%
Perforation corrosion	Very severe corrosion or perforation	More than 85%

### 3.2 Corrosion conditions in casing-damaged wells

**Figure 1:**
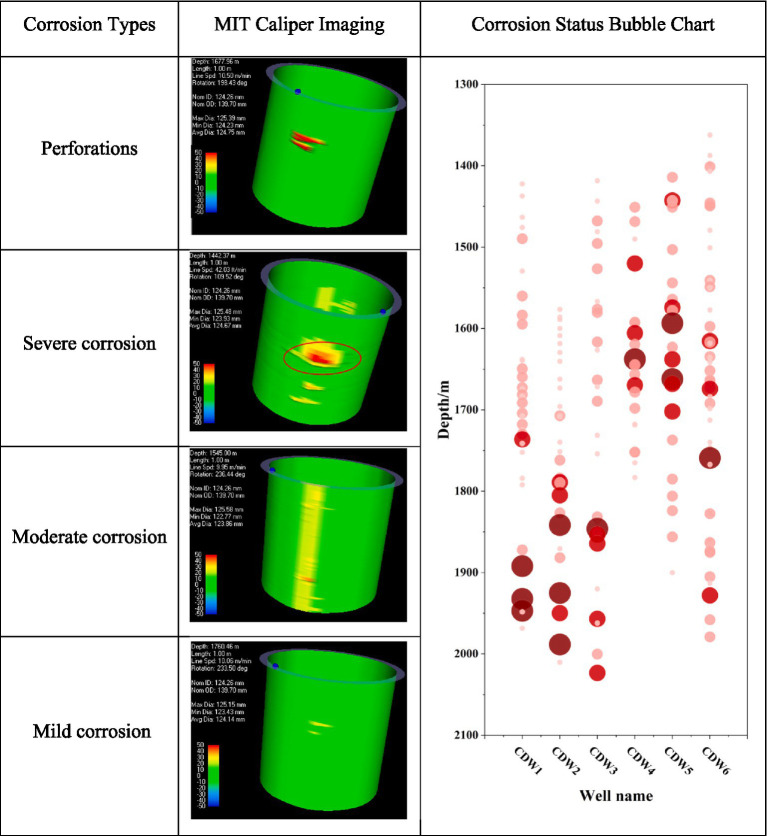
Multi-Finger Imaging Tool (MIT) caliper imaging at different corrosion levels. Perforations: 1347.89 m, 1576.23 m; Severe corrosion: 1485.00–1499.00 m and 1506.00–1520.00 m; moderate corrosion: 1338.26 m and 1451.00–1457.00 m; mild corrosion: 1365.00–1380.00 m and 1410.00–1425.00 m.

For the six casing-damaged wells, overall corrosion was observed in the interval between 1,400 m and 2,100 m (Figure 1), corresponding to the Luohe and Andingzhiluo formations. However, the locations of severe corrosion varied among the wells. In wells CDW1, CDW2, and CDW3, perforations and severe corrosion primarily occurred between 1800 m and 2000 m, whereas in CDW4 and CDW5, these issues mainly appeared between 1,500 m and 1700 m. CDW6 exhibited the widest span of corrosion, while CDW4 had the smallest corrosion zone and the overall lowest degree of corrosion. Although CDW2 had the highest overall corrosion level, there was no significant difference when compared with the other wells (with the exception of CDW4).

### 3.3 Microbial community diversity in produced fluids

**Figure 2:**
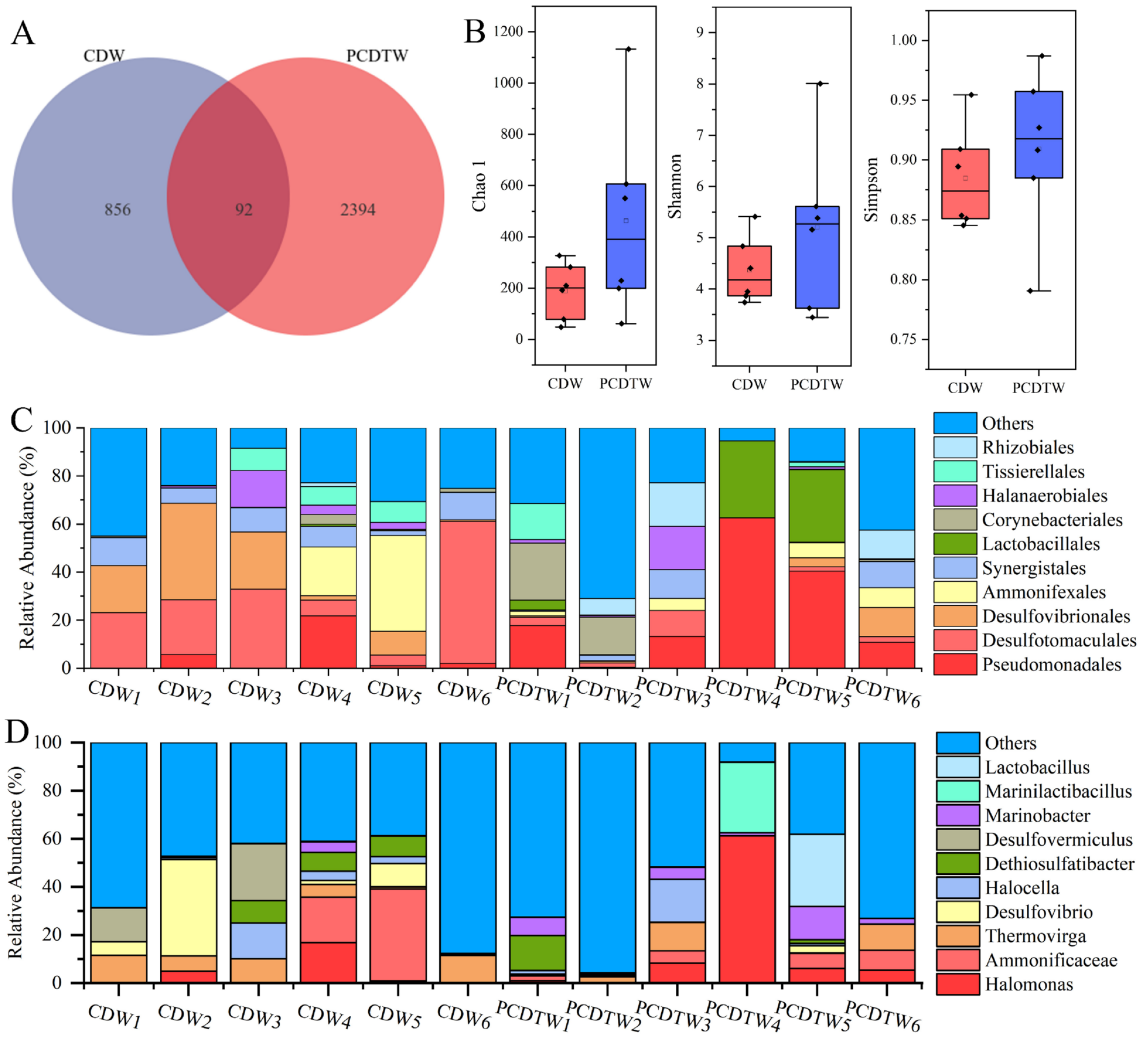
**(A)** Venn diagram of ASVs from production fluids of damaged and normal wells. **(B)** Alpha diversity index analysis. **(C)** Taxonomic composition at the order level. **(D)** Taxonomic composition at the genus level.

Alpha diversity index analyses revealed that the Chao1, Shannon, and Simpson indices for the CDW group were lower than those for the PCDTW group (Figure 2B). This indicates that the microbial species richness and diversity in the produced fluids from casing-damaged wells are lower than those in fluids from normally operating wells. Analysis using Venn diagrams showed that the PCDTW group possessed 2,394 unique ASVs, while the CDW group had 856 unique ASVs, with only 92 ASVs shared between the two groups (Figure 2A).

Combining the diversity analyses and the ASV Venn diagram results, it was evident that the microbial community within the casing-damaged wells exhibited reduced biodiversity, and SRB dominated the community, suppressing the growth of other functional microorganisms. Li et al. (2016) investigated microbial diversity and composition in high-temperature oil reservoirs from Jiangsu Oilfield with high sulfate content and known corrosion, and found that *Desulfotignum* spp. was the dominant SRB in both samples, with low overall SRB diversity. Similarly, Lv et al. (2022) demonstrated that mixed microbial communities of SRB and iron-oxidizing bacteria (IOB) produced synergistic effects that exacerbated localized corrosion of X65 steel compared to single-species systems, with SRB gradually evolving into the dominant group. SRB may inhibit other microbial groups by releasing antimicrobial substances (e.g., hydrogen sulfide), while the sulfides produced during their metabolism can react with heavy metals (e.g., Fe^2+^ forming FeS precipitates), indirectly altering metal ion concentrations in the environment and affecting the activity of other microorganisms, thereby establishing dominance within the community (Anandkumar et al., 2016; Lou et al., 2021).

### 3.4 Composition of microbial communities in produced fluids

Analysis of the microbial community composition revealed that SRB were highly abundant in the produced fluids from the casing-damaged wells (Figure 2C). In wells CDW1, CDW2, and CDW3, SRB were predominantly represented by the orders Desulfovibrionales and Desulfotomaculales. In contrast, wells CDW4 and CDW5 mainly harbored SRB belonging to the order Ammonifexales, while in CDW6, more than half of the SRB were classified as Desulfotomaculales. Given that not all genera within the orders Desulfovibrionales and Desulfotomaculales are confirmed SRB, further community composition analysis was conducted at the genus level (Figure 2D). In wells CDW1, CDW2, and CDW3, SRB were predominantly represented by *Desulfovibrio* and *Desulfovermiculus* (both belonging to the order Desulfovibrionales). Wells CDW4 and CDW5 were primarily dominated by *Ammonificaceae* (belonging to the Ammonifexales order). Members of *Ammonificaceae* are widely distributed in deep subsurface environments and are capable of utilizing hydrogen as an electron donor to perform sulfate reduction, producing H₂S (Mura et al., 2024). In contrast, the majority of the microbial community in CDW6 was classified as others, with *Thermovirga* being the identified SRB.

In the wells after casing repair, the dominant microbial groups were Pseudomonadales and Lactobacillales. Members of Pseudomonadales are commonly detected in oil reservoir samples. For instance, one study reported that following the injection of nutrients (carbon and nitrogen sources, along with inorganic salts and trace elements) into well S12-4, samples extracted between 0 and 17 months post-injection were dominated by species of *Pseudomonas* (order Pseudomonadales) and *Marinobacter* (Xingbiao et al., 2015). In another study by Fu et al. (2025), an oil reservoir in the Daqing Oilfield experienced an average oil production increase of 218.6–221.4% after nutrient injection, and 1 year after the cessation of injection, a stable microbial community dominated by *Pseudomonas* was established. Additionally, Pedersen and Hermansson (1991) demonstrated that two marine isolates of *Pseudomonas* spp. inhibited steel corrosion.

### 3.5 Comparison of microbial communities between casing-damaged and repaired wells

**Figure 3:**
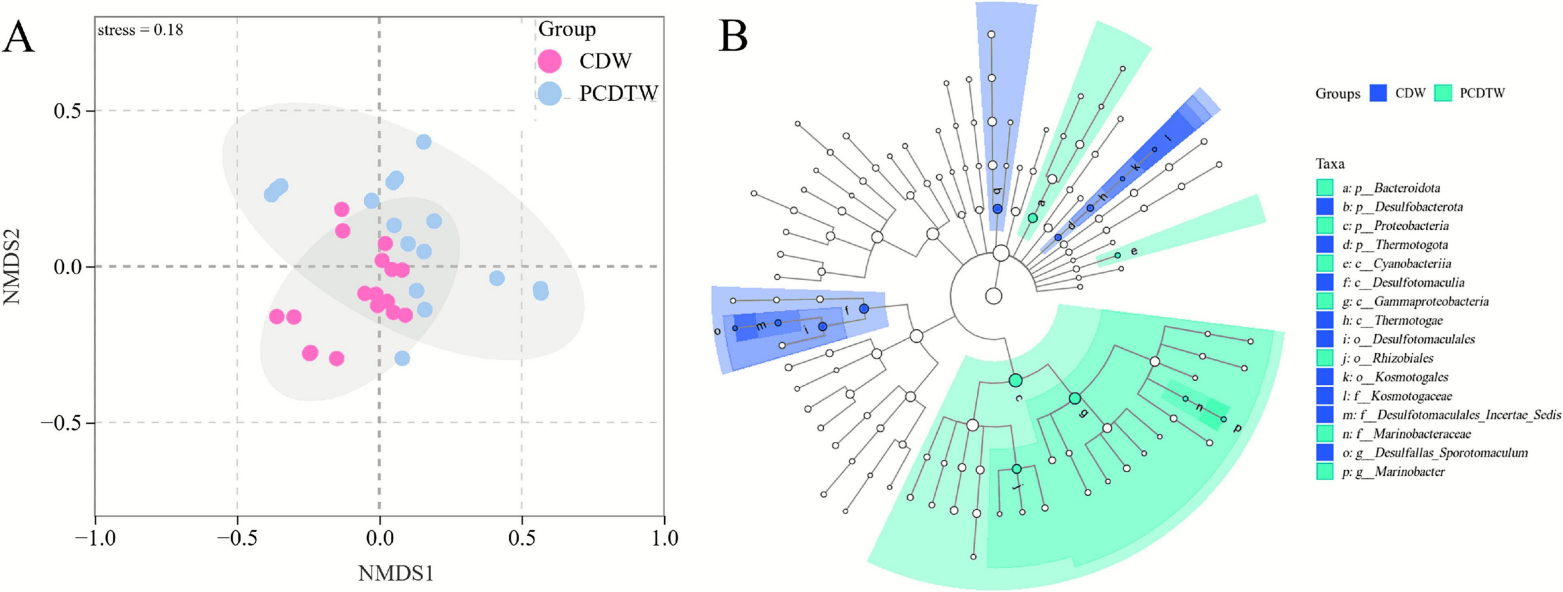
**(A)** Results of the Bray–Curtis NMDS analysis. **(B)** LEfSe (LDA effect size) analysis of microbial communities.

As shown in the Figure 3A, a clear separation is observed between the CDW and PCDTW groups. The CDW group is tightly clustered in the lower left section, indicating high within-group similarity, while the PCDTW group is relatively dispersed in the upper right region. The stress value is 0.18, which is below the 0.2 threshold, demonstrating that the NMDS plot provides a reliable representation of the dissimilarities among samples.

LEfSe (LDA Effect Size) analysis, which integrates the non-parametric Kruskal–Wallis and Wilcoxon rank-sum tests with linear discriminant analysis (LDA) effect size measurement (Segata et al., 2011), was employed to identify robust differential taxa between groups as biomarkers. According to the LEfSe analysis, the significantly enriched microorganisms in the PCDTW group included members of the Pseudomonadales such as Marinobacteraceae *Marinobacter* and bacteria within the order Rhizobiales (Figure 3B). In the LEfSe results, Marinobacteraceae *Marinobacter* was found to be significantly enriched in the PCDTW group. Species of *Pseudomonas* are widely distributed in various oilfield ecosystems and possess strong crude oil degradation capabilities (Pannekens et al., 2019). *Marinobacter* strains have been detected in deep-sea samples and core samples from oil drilling platforms; some studies have even proposed *Marinobacter* as a potential indicator for identifying crude oil reservoirs in different deep-sea regions of the Red Sea (Jamal, 2020). The presence of these taxa in the PCDTW group suggests that the oilfield holds considerable potential for enhancing oil recovery through the injection of carbon and nitrogen nutrients to stimulate the indigenous microbial community.

In contrast, the CDW group was characterized by a significant enrichment of microorganisms including *Desulfallas Sporotomaculum* of the order Desulfotomaculales and members of the family *Kosmotogaceae* within the phylum Thermotogae. In a 100-day experiment examining the interactions among supercritical CO₂, water, coal, and microorganisms, *Desulfallas Sporotomaculum* (exceeding 9% abundance) was identified as one of the dominant microorganisms. In a study of subsurface microbial communities hosted by basalt in the Surtsey volcanic island in Iceland—where temperatures exceeded 120 °C—Bergsten et al. (2021) reported that *Desulfallas Sporotomaculum*, along with *Thermaerobacter*, *Thioalkalimicrobium*, and *Sulfurospirillum*, represented indigenous *in situ* microorganisms. The family *Kosmotogaceae* comprises anaerobic heterotrophs primarily distributed in high-temperature environments such as oilfields and geothermal springs. For instance, *Kosmotoga olearia* gen. nov., sp. nov. was isolated from petroleum production fluids on the Troll B oil platform in the North Sea; its growth is stimulated by sulfate and thiosulfate, although it does not produce hydrogen sulfide (DiPippo et al., 2009).

### 3.6 Microbial functional prediction

**Figure 4:**
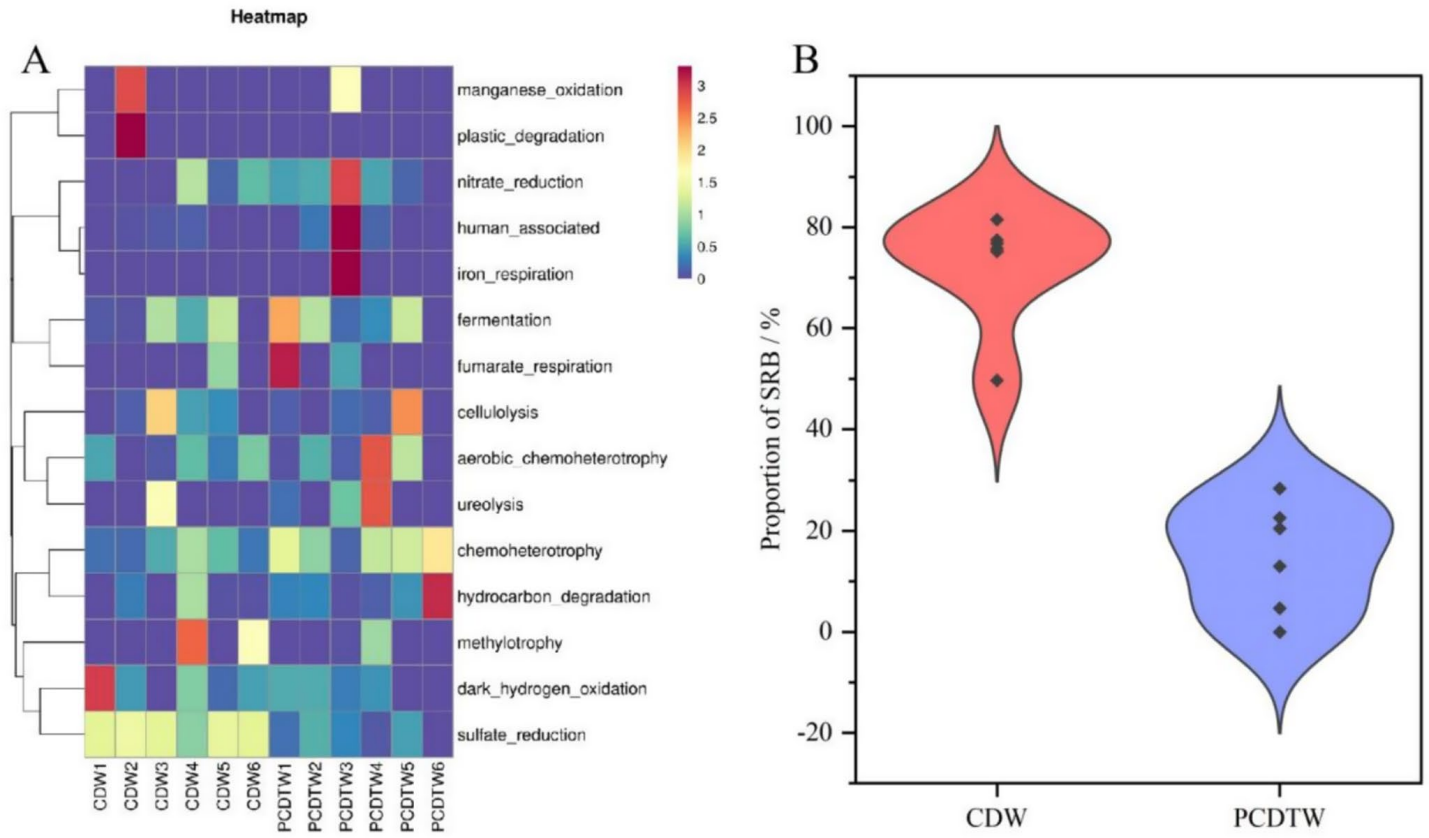
**(A)** Functional annotation of microbial communities in production fluids from casing-damaged wells. **(B)** Distribution of SRB proportions in casing-damaged wells.

The functional prediction of the microbial communities in the produced fluids was performed using FAPROTAX and further validated by quantitative measurements of the absolute abundance of SRB and the concentration of their metabolic product, sulfide. The proportion of SRB in the CDW group was significantly higher than in the PCDTW group (Figure 4A). Quantitative results of the absolute SRB counts (Table 4) revealed that the SRB concentration in the produced fluids of the CDW group reached 10^5^–10^6^ pcs /mL, which is nearly three orders of magnitude higher than that in the PCDTW group (10^2^–10^3^ pcs /mL). Concurrently, the sulfide content was markedly elevated in the CDW group, providing direct evidence that the SRB population in these wells not only dominated in abundance but also exhibited vigorous metabolic activity of sulfate reduction, leading to substantial production of hydrogen sulfide—a key agent in corrosion.

**Table 4:** SRB content and sulfide content in different wells.

Well name	SRB content (pcs/mL)	Sulfide content (mg/L)
CDW-1	5.04E+06	463.04
CDW-2	4.02E+06	481.45
CDW-3	4.27E+06	419.17
CDW-4	2.60E+05	163.47
CDW-5	5.50E+06	343.75
CDW-6	4.00E+06	410.42
PCDTW-1	5.30E+02	63.48
PCDTW-2	3.70E+03	38.76
PCDTW-3	7.57E+02	36.38
PCDTW-4	1.24E+03	19.08
PCDTW-5	2.83E+03	40.07
PCDTW-6	1.30E+02	5.11

The absolute dominance of SRB in the CDW group likely restricts the ecological niche for other functional microorganisms via competitive inhibition. In contrast, functions related to chemical heterogeneity, hydrocarbon degradation, and fermentation were slightly more pronounced in the PCDTW group than in the CDW group. This may be attributed to the creation of an ecological opportunity for other functional microbiota following the effective suppression of SRB after remedial operations.

In the study by Wang et al. (2025), microorganisms possessing a range of anaerobic hydrocarbon degradation genes were considered “versatile” microbes within the reservoir, and the findings indicated that these versatile microbial communities included various SRB. SRB can utilize molecular hydrogen, low-molecular-weight alcohols, low-molecular-weight fatty acids (such as acetate, lactate, and pyruvate), higher fatty acids, aliphatic hydrocarbons, and monoaromatic compounds as carbon sources (Muyzer and Stams, 2008). The high abundance of SRB and their robust metabolic activity detected in the CDW group in this study suggest that the abundance of functional genes associated with the anaerobic degradation of hydrocarbons by SRB is a key factor supporting their dominance in the biofilm system on the wellbore walls.

Statistics show that in the casing-damaged wells, microorganisms associated with sulfate reduction account for approximately 80% of the community, whereas in the wells after repair, this proportion is generally below 40% (Figure 4B). This finding, particularly when combined with the notable reduction in both the absolute abundance of SRB and sulfide concentration, strongly indicates that the remedial operation effectively suppresses the presence and activity of SRB in the produced fluids. Consequently, the SRB content—encompassing both relative abundance and absolute quantity—in the produced fluid microbial community can serve as a key bio-indicator for assessing wellbore integrity. A consistently low level of SRB suggests a favorable wellbore condition with a controlled corrosion risk.

**Figure 5:**
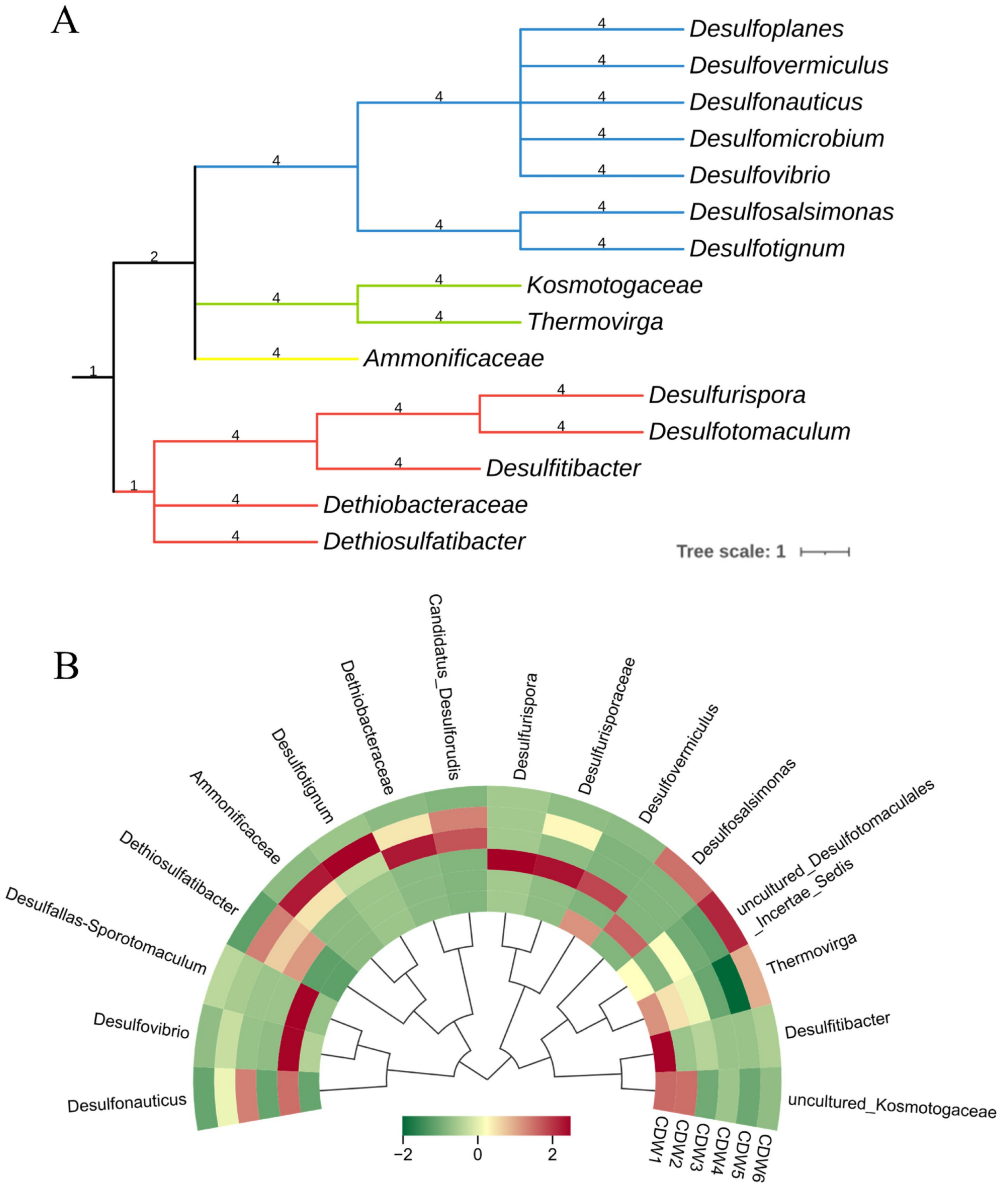
**(A)** Phylogenetic tree of SRB in production fluid samples. **(B)** Genus-level distribution of SRB in casing-damaged wells.

A phylogenetic tree was constructed to depict the evolutionary relationships within the same lineage, which helped identify the most closely related species. The phylogenetic analysis revealed that the SRB present in the produced fluids from the casing-damaged wells clustered into four major branches, representing the phyla Proteobacteria, Bacillota, Thermotogata, and Thermodesulfobacteriota, which are depicted in orange, purple, green, and pink, respectively (Figure 5A). Within Bacillota, the taxa were mainly divided into the classes Tissierellia and Clostridia. The Thermotogata branch was primarily subdivided into Thermotogota and Synergistota. The Thermodesulfobacteriota branch was mainly divided into Desulfobacterales and Desulfovibrionales, whereas Proteobacteria was represented solely by *Ammonificaceae*. In terms of species number, Bacillota and Thermodesulfobacteriota contained the greatest diversity. These results are consistent with previous studies. In oil reservoirs, certain SRB-associated taxa related to Proteobacteria, Bacillota, Thermodesulfobacteria, Nitrospira, Euryarchaeota, and Crenarchaeota are most frequently detected, including genera such as *Desulfovibrio*, *Desulfomicrobium*, *Desulfobacterium*, *Desulfococcus*, *Desulfobulbus*, *Desulfotomaculum*, *Thermodesulfobacterium*, *Thermodesulfovibrio*, and *Archaeoglobus* (Wang et al., 2025).

### 3.7 Correlation analysis between casing damage location and SRB

Based on the clustering heatmap, the distribution of SRB across different samples was observed (Figure 5B). Overall, CDW1 and CDW2 exhibited high similarity, as did CDW4 and CDW5. In CDW1, the predominant microorganism was *Desulfitibacter*; in CDW2, *Desulfovibrio* and *Desulfallas Sporotomaculum* were most abundant; in CDW3, *Desulfurispora* and members of the *Desulfurisporaceae* family dominated; in CDW4, the dominant taxa belonged to the *Dethiobacteraceae*; in CDW5, *Desulfotignum* and *Ammonificaceae* prevailed; and in CDW6, the predominant group was classified as *uncultured Desulfotomaculales Incertae Sedis*.

By combining the bubble chart of corrosion hotspots with the microbial community composition data from different wells, it was found that the depth of the corrosion hotspots may be related to the composition of the microbial communities in the produced fluids. Depth is one of the critical factors affecting the composition of oil reservoir microbial communities; for every 100 m increase in depth, the temperature rises by approximately 2–3 °C, and pressure correspondingly changes. Song et al. (2021) suggested that the geothermal gradient, oxygen depletion, and fluid turbulence are the primary factors influencing the vertical distribution of microbial communities, proposing an ecosystem boundary for wellbore biofilms at around 1,000 m depth. Specifically, in wells CDW1, CDW2, and CDW3, perforations and severe corrosion occurred primarily between 1800 m and 2000 m, with the SRB community mainly comprising *uncultured Kosmotogaceae*, *Thermovirga*, and *uncultured Desulfotomaculales Incertae Sedis*. The first two microorganisms belong to the phylum Thermotogata, a group known for its thermophilic characteristics and typically found in high-temperature environments such as hydrothermal vents, marine sediments, crustal fluids, deep aquifers, oil reservoirs, and anaerobic engineered systems (Gupta and Bhandari, 2011).

In contrast, in wells CDW4 and CDW5, where perforations and severe corrosion were mainly observed between 1,500–1700 m depth, the SRB community was predominantly composed of *Ammonificaceae*, *Desulfotignum*, *Candidatus Desulforudis*, and *Dethiobacteraceae*, taxa affiliated with the phylum Proteobacteria and Bacillota. Zhou et al. (2023) reported that in medium- to low-temperature oil reservoirs, the core sulfate-reducing prokaryote (SRP) groups include *Desulfomicrobium*, *Desulfovibrio*, and *Desulfotomaculum*, whereas in high-temperature reservoirs, core groups include the archaeal genus *Archaeoglobus*, as well as *Thermodesulfobacterium* and *Thermodesulfovibrio*. Moreover, Li et al. (2017) investigated the biocorrosion potential of produced water samples from a high-temperature reservoir (79.6 °C, 1809 m depth) and found that the dominant microorganisms in the produced fluids varied with temperature: at 37 °C, SRB closely related to *Desulfotignum*, *Desulfobulbus*, and *Desulfovibrio* were observed, while at higher temperatures, the predominant SRB were associated with *Desulfotomaculum* and *Archaeoglobus*.

In summary, the comprehensive analysis of corrosion conditions and microbial community composition across different wells indicates that environmental parameters such as well depth, temperature, and pressure at the casing damage locations have a significant influence on the structure of the microbial communities in the produced fluids.

### 3.8 Correlation analysis between the degree of casing damage and SRB

**Figure 6:**
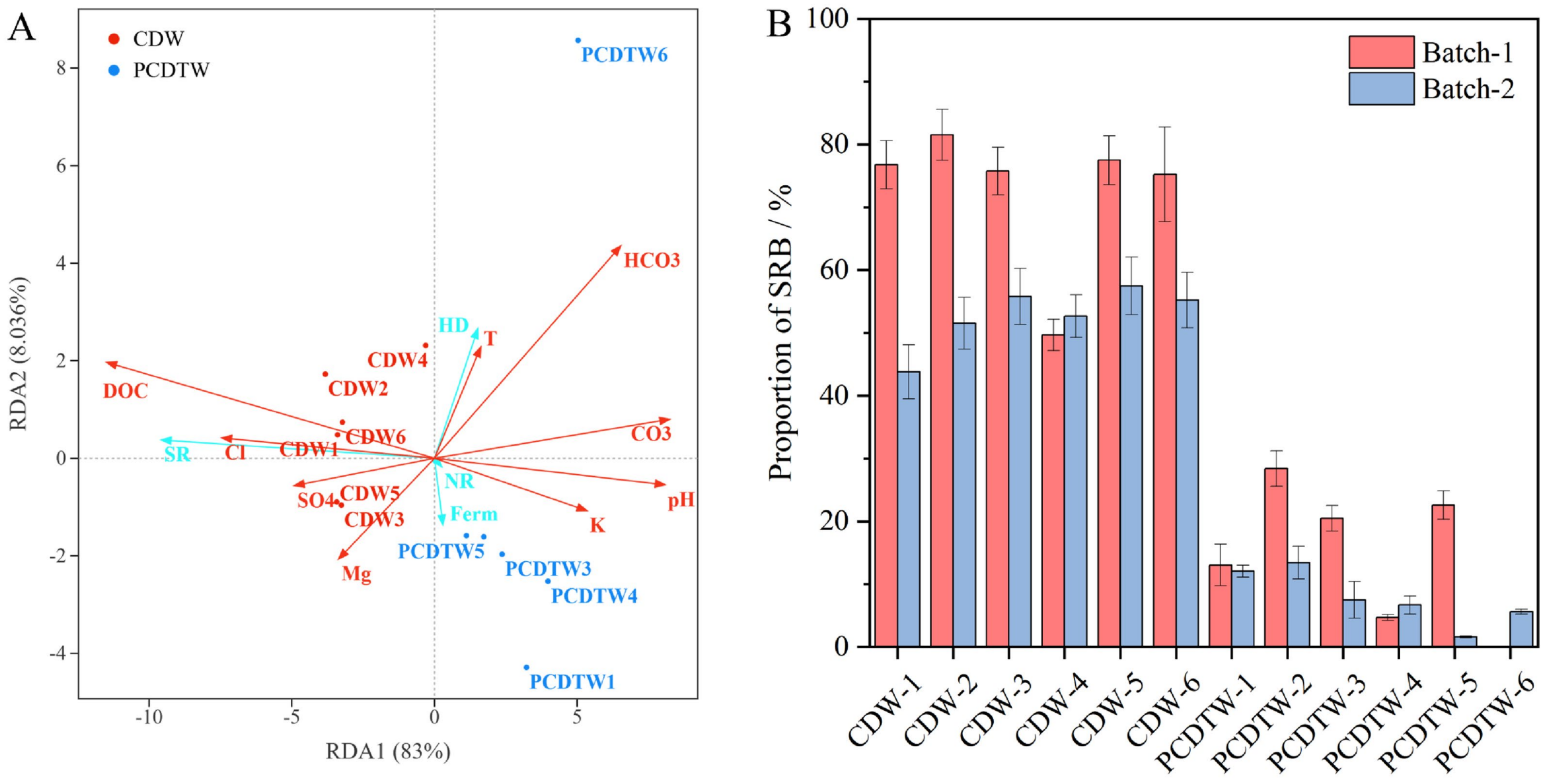
**(A)** Relative abundance of sulfate-reducing microorganisms in production fluids and results of RDA analysis. The red line represents the environmental factor, and the blue line represents the microbial functional type. DOC, degree of corrosion; Cl, Cl ^−^; SO4, SO_4_^2−^; Mg, Mg^2+^; K, K ^+^; CO3, CO_3_^2−^; HCO3, HCO_3_^−^; T, Temperature, °C; SR, Sulfuric acid reduction; NR, Nitrate reduction; Ferm, fermentation; HD, degradation of hydrocarbons; **(B)** column diagram of the proportion of SRB in the two batches of liquid production (Batch-1: 2024.08; Batch-2: 2024.11).

The chemical composition of formation water directly affects the SRB community, particularly the availability of electron acceptors and donors. In subsurface oil reservoirs with low redox potential, electron acceptors such as oxygen, nitrate, and ferric iron are typically limited. For instance, SRB adapt to the reservoir environment by reducing sulfate to hydrogen sulfide via a series of enzymatic reactions. Redundancy analysis (RDA) was conducted on the ionic content of the produced fluids, environmental parameters, the extent of corrosion, and the microbial communities. The microbial community was functionally partitioned into SRB, nitrate-reducing bacteria, fermentative bacteria, and hydrocarbon-degrading bacteria. Together, the first two RDA axes explained 91.036% of the total variance (Figure 6). Chloride (Cl^−^), sulfate (SO₄^2−^), and the degree of corrosion showed a positive correlation with the abundance of SRB, whereas the ratios of potassium to sodium (K/Na^+^) and carbonate (CO₃^2−^) exhibited a negative correlation with SRB. Temperature and pressure often interact synergistically with other environmental factors to influence microbiologically influenced corrosion (MIC). In this study, the impact of temperature and pressure on the corrosion severity and the abundance of SRB was relatively minor, which can be attributed to the limited variation in bottom-hole depth and temperature across the samples (Table 1). In contrast, pH exhibited a negative correlation with both SRB abundance and the degree of corrosion. pH value can modulate the corrosion type and rate by regulating the microbial community structure and the electrochemical environment (Li et al., 2025). In low-pH (acidic) environments, the high concentration of hydrogen ions facilitates the cathodic hydrogen evolution reaction, thereby accelerating corrosion. Furthermore, acidic conditions are more favorable for the growth of acid-producing bacteria and sulfur-oxidizing bacteria, which are typically associated with uniform corrosion (Alaba and Odeyemi, 2024).

The SRB were negatively correlated with other microbial groups. A competitive relationship exists between nitrate-reducing bacteria and SRB, as both groups consume common nutrients, such as electron donors (e.g., hydrogen and organic compounds) and electron acceptors (e.g., nitrate and sulfate), thereby restricting each other’s growth and metabolic activity. Conversely, fermentative bacteria and SRB often engage in a synergistic relationship; fermentative bacteria convert hydrocarbons into secondary metabolites such as volatile fatty acids, methylated compounds, acetate, carbon dioxide, and hydrogen. Acetate and propionate can serve as both electron donors and carbon sources for SRB, while formate may function similarly to hydrogen as an electron donor. In this study, SRB similarly suppressed the proportion of fermentative bacteria. Liu et al. (2024), through surface analysis and electrochemical measurements, investigated the individual roles of iron-oxidizing bacteria (IOB) and SRB in the microbiologically influenced corrosion of carbon steel pipelines, and also observed that SRB became the dominant microorganisms during the later stages of corrosion.

Figure 6A shows that the more severe the casing damage, the higher the proportion of SRB in the produced fluids. This finding is consistent with previous studies (Rajala et al., 2015). For example, Yuan et al. (2023) analyzed the causes of severe localized internal corrosion in crude oil pipelines after short-term operation through thickness measurements, microbial analysis, SEM, EDS, and XRD, and found that extensive corrosion pits with thick and complex corrosion products/deposits formed at the pit edges contained abundant SRB. In the study by Nasser et al. (2021), X65 steel samples used in corrosion simulation experiments demonstrated that samples with higher sulfate concentrations harbored a greater abundance of SRB, as evidenced by SEM and confocal laser scanning microscopy (CLSM). Similarly, Zhou et al. (2023) reported that the corrosion rate on carbon steel surfaces in production water was higher than in controls, and that this rate correlated positively with the abundance of SRP. Liu et al. (2022) further demonstrated that both the uniform and localized corrosion rates were proportional to the initial concentration and survival numbers of SRB.

### 3.9 Long-term stability analysis of microbial-based monitoring method

To validate the stability of the microbial communities in production fluids, we performed a second sampling event after a three-month interval. The results indicated an overall relative decrease in SRB in the second batch compared to the first batch (Figure 6B). Indeed, reservoir microbial communities are susceptible to anthropogenic interventions. For instance, in the Halfdan oil field, as reservoir production and development progressed, the intrinsic community successively shifted from anaerobic taxa to fast-growing opportunists characterized by metabolic traits such as nitrate reduction and sulfur oxidation (Vigneron et al., 2017). Although microbial communities varied over time, the relative abundance of SRB in the CDW group remained significantly higher than that in the PCDTW group. This suggests that SRB can serve as a reliable and stable indicator for long-term monitoring of casing damage in oilfield production operations.

**Figure 7:**
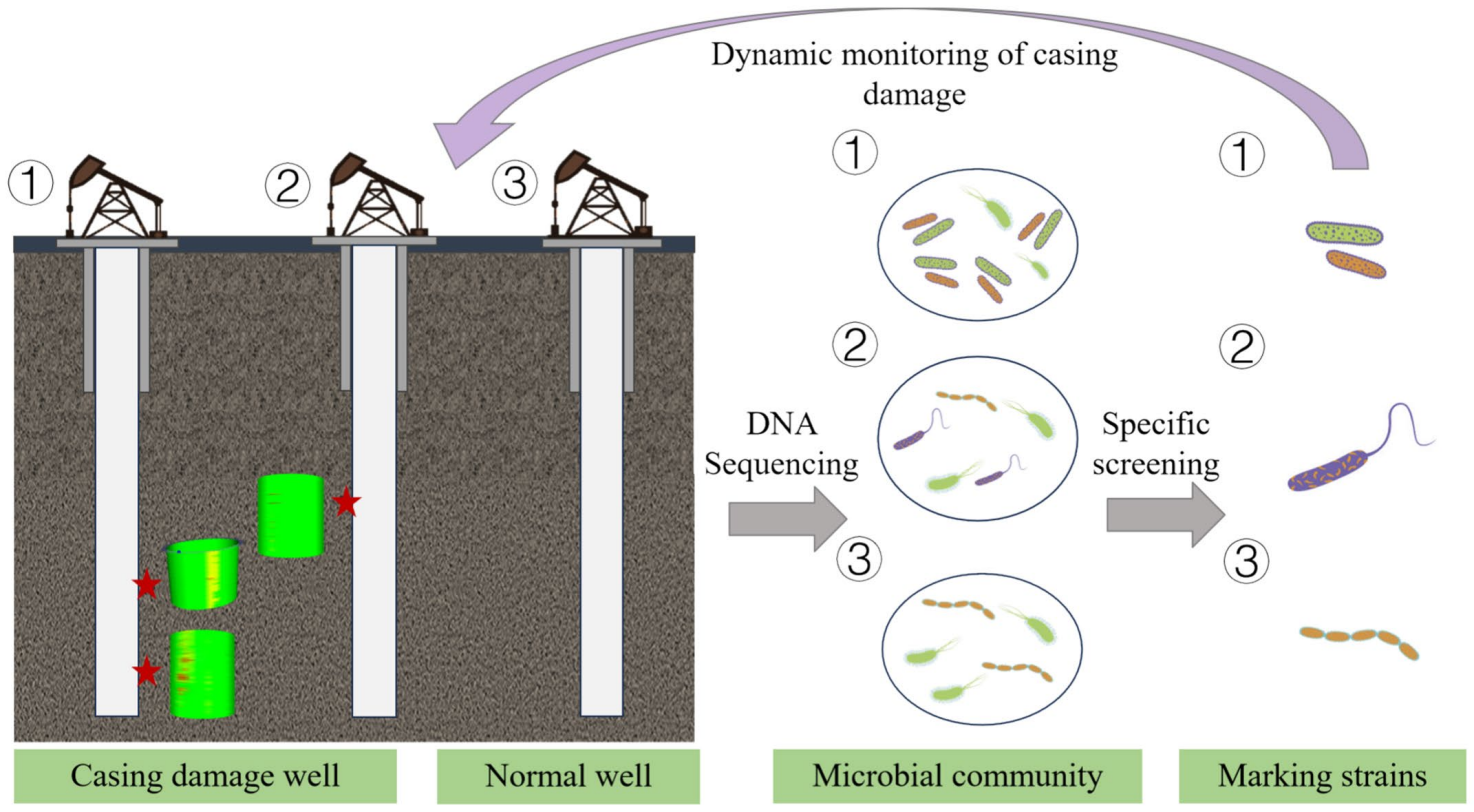
Microbial early-warning model for casing damage.

Overall, the research findings suggest that a dynamic monitoring system based on SRB can serve as an effective auxiliary method for assessing casing damage. Compared with traditional corrosion monitoring techniques (such as mechanical caliper logging, ultrasonic testing, and coupon methods) that require long-term operational investment, the novel microbial DNA sequencing approach exhibits significant advantages. It offers low per-sample costs, non-destructive sampling, and online monitoring capabilities, thereby enabling real-time tracking of the dynamic succession of corrosion-related microbial communities to provide early warning of corrosion risks. This technology has the potential to shift the monitoring of casing-damaged wells from a reactive maintenance approach to a proactive “biological signal early warning and remediation intervention” strategy (Figure 7), thereby advancing the petroleum industry’s monitoring system toward biologically intelligent detection methods.

However, despite its promising prospects, the technology’s limitations in geographical and environmental representativeness cannot be overlooked. All samples in this study were sourced exclusively from the specific operational area of Taibailiang in the Changqing Oilfield. Significant heterogeneity exists among different reservoirs regarding key environmental parameters such as temperature, salinity, pressure, and indigenous microbial community structure. Consequently, the microbial corrosion dynamics model and early-warning thresholds established based on findings from this particular location may not be linearly extrapolated to other petroleum production systems, such as high-salinity reservoirs, offshore oilfields, or those with distinct geological backgrounds. This spatial sampling constraint, coupled with ecological variability, collectively constitutes a major limitation to the universal applicability of the technology. Thereby, it somewhat undermines its external validity and feasibility for broad industry adoption. Future research urgently requires cross-regional validation across diverse reservoir types to establish a more universally applicable biomarker system and calibration model.

## 4 Conclusion

This study is the first to combine MIT–MTT integrated logging technology with 16S rRNA gene analysis to analyze the relationship between casing damage severity and microbial community composition in wells. The results indicate that the location and degree of casing damage are the primary factors influencing the microbial composition of the produced fluids. In regions with severe corrosion at greater depths, there is an increased abundance of thermophilic SRB such as those within the phylum Thermotogata. SRB are identified as the principal microbial agents responsible for the metal corrosion of casings, and wells treated with secondary sealing exhibit a significantly reduced proportion of SRB. A positive correlation was observed between the degree of casing damage and the abundance of SRB. By integrating the corrosion status of production wells with the dynamic changes in the microbial community of the produced fluids, this study proposes a microbial dynamic monitoring technique capable of predicting the locations and extent of casing damage.

## Data Availability

The data presented in the study were deposited to the Sequence Read Archive of the NCBI database under accession no. PRJNA1359529.

## Footnotes

1
http://www.zoology.ubc.ca/louca/FAPROTAX/

2
https://bugbase.cs.umn.edu/
