# Supplementary material for: Research on casing damage risk early warning technology based on MIT-MTT logging and 16S rRNA gene analysis
Source: Front Microbiol. 2025 Dec 15;16:1708148. doi: 10.3389/fmicb.2025.1708148 (PMC12747668; doi:10.3389/fmicb.2025.1708148)
Supplement: Supplementary file 1 [file Table_1.docx]

# Research on Casing Damage Risk Early Warning Technology Based on MIT-MTT Logging and 16S DNA Sequencing

Shuoliang Wang^a,1,*^,Shiqi Wang^a,1^, Congcong Li^b^, Changhao Zhou^a^, Liangliang Jiang ^c,*^

*^a^School of Energy, Faculty of Engineering, China University of Geosciences, Beijing 100083, PR China*

*^b^College of Civil Engineering and Architecture, Shandong University of Aeronautics, Binzhou City, Shandong Province 256600, PR China*

*^c^Department of Chemical and Petroleum Engineering, University of Calgary, Canada*

*^1^ Co-first authors*

*Corresponding authors:

Shuoliang Wang, [wangshuoliang@cugb.edu.cn](mailto:wangshuoliang@cugb.edu.cn);

Liangliang Jiang, [jial@ucalgary.ca](mailto:jial@ucalgary.ca)

Table S1. Microbial species information in the blank control samples

| Phylum | Class | Order | Family | Genus | Species |
| --- | --- | --- | --- | --- | --- |
| Pseudomonadota | Gammaproteobacteria | Enterobacterales | Enterobacteriaceae | Escherichia-Shigella | Unassigned |
| Bacillota | Clostridia | Christensenellales | Christensenellaceae | Christensenellaceae_genus | Unassigned |
| Pseudomonadota | Gammaproteobacteria | Enterobacterales | Yersiniaceae | Serratia | Unassigned |
| Pseudomonadota | Gammaproteobacteria | Burkholderiales | Comamonadaceae | Unassigned | Unassigned |
| Bacteroidota | Bacteroidia | Sphingobacteriales | Lentimicrobiaceae | Lentimicrobium | bacterium_sp. |
| Bacillota | Clostridia | Lachnospirales | Lachnospiraceae | [Eubacterium]_xylanophilum_group | uncultured_bacterium |
| Actinomycetota | Coriobacteriia | Coriobacteriales | Coriobacteriales_Incertae_Sedis | Raoultibacter | uncultured_bacterium |
| Actinomycetota | Actinobacteria | Micrococcales | Intrasporangiaceae | Unassigned | Unassigned |
| Bacillota | Bacilli | Bacillales | Bacillaceae | Bacillus | Unassigned |
| Bacteroidota | Bacteroidia | Bacteroidales | Prevotellaceae | Prevotellaceae_UCG-001 | uncultured_bacterium |
| Pseudomonadota | Alphaproteobacteria | Rhodobacterales | Paracoccaceae | Unassigned | Unassigned |
| Pseudomonadota | Gammaproteobacteria | Burkholderiales | Comamonadaceae | Aquabacterium | Unassigned |
| Spirochaetota | MVP-15 | MVP-15_order | MVP-15_family | MVP-15_genus | uncultured_Spirochaetes |
| Actinomycetota | Actinobacteria | Mycobacteriales | Corynebacteriaceae | Corynebacterium | Unassigned |
| Actinomycetota | Actinobacteria | Propionibacteriales | Propionibacteriaceae | Propioniciclava | uncultured_bacterium |
| Actinomycetota | Actinobacteria | Propionibacteriales | Propionibacteriaceae | Cutibacterium | Unassigned |
| Bacillota | Clostridia | Lachnospirales | Lachnospiraceae | Butyribacter | uncultured_bacterium |
| Pseudomonadota | Gammaproteobacteria | Burkholderiales | Rhodocyclaceae | Unassigned | Unassigned |
| Actinomycetota | Actinobacteria | Mycobacteriales | Corynebacteriaceae | Lawsonella | Unassigned |
| Pseudomonadota | Gammaproteobacteria | Burkholderiales | Burkholderiaceae | Ralstonia | Unassigned |
| Bacillota | Bacilli | Staphylococcales | Staphylococcaceae | Staphylococcus | Unassigned |
| Pseudomonadota | Alphaproteobacteria | Rhodobacterales | Paracoccaceae | Paracoccus | Unassigned |
| Bacteroidota | Bacteroidia | Bacteroidales | Bacteroidaceae | Bacteroides | Bacteroides_caecigallinarum |
| Bacillota | Clostridia | Peptostreptococcales-Tissierellales | Anaerovoracaceae | Family_XIII_AD3011_group | Unassigned |
| Pseudomonadota | Alphaproteobacteria | Hyphomicrobiales | Xanthobacteraceae | Bradyrhizobium | Unassigned |
| Bacillota | Clostridia | Oscillospirales | Ruminococcaceae | Unassigned | Unassigned |
| Pseudomonadota | Alphaproteobacteria | Hyphomicrobiales | Hyphomicrobiaceae | Filomicrobium | uncultured_compost |
| Pseudomonadota | Alphaproteobacteria | Sphingomonadales | Sphingomonadaceae | Sphingomonas | Unassigned |
